# Quality Attributes of Refrigerated Barhi Dates Coated with Edible Chitosan Containing Natural Functional Ingredients

**DOI:** 10.3390/foods11111584

**Published:** 2022-05-28

**Authors:** Kashif Ghafoor, Fahad Y. Al-Juhaimi, Elfadil E. Babiker, Isam A. Mohamed Ahmed, Syed Ali Shahzad, Omer N. Alsawmahi

**Affiliations:** Department of Food Science and Nutrition, College of Food and Agricultural Sciences, King Saud University, P.O. Box 2460, Riyadh 11451, Saudi Arabia; faljuhaimi@ksu.edu.sa (F.Y.A.-J.); ebabiker.c@ksu.edu.sa (E.E.B.); iali@ksu.edu.sa (I.A.M.A.); syedalishahzad@gmail.com (S.A.S.); omernasser2009@hotmail.com (O.N.A.)

**Keywords:** Barhi dates, *Phoenix dactylifera*, chitosan, orange peel, olive cake, coating, quality attributes, scanning electron microscopy, surface structure

## Abstract

Edible chitosan coatings with natural functional ingredients were used to preserve quality attributes of fresh Barhi date fruit. Fruits were coated with chitosan and/or 1 and 2% olive cake extract (OCE) or orange peel extract (OPE). Both coated and uncoated fruits were stored at 4 °C for 4 weeks. A slight decrease in the pH and increase in acidity with storage was observed. However, when chitosan was mixed with OCE or OPE, an increase in pH was observed with a concomitant decrease in acidity. The phenolic content of the samples was decreased with time. However, coating the date with OCE or OPE significantly (*p* ≤ 0.05) increased the total phenolic with a concomitant increase in radical scavenging activity. The textural properties, particularly hardness, were better preserved in case of coated dates. The sensory evaluation data showed non-significant changes in the acceptability of the Barhi dates throughout the storage period. Chitosan-coating significantly (*p* ≤ 0.05) inhibited mold growth over time. Scanning electron microscope (SEM) imaging showed difference among different coatings. According to principal component analysis (PCA), OCE and OPE were found to have protective effects on fruit quality.

## 1. Introduction

Date (*Phoenix dactylifera*) palm is one of the essential subsistence crops and a major fruiting tree in the Middle East and some other countries [1]. This tree bears a climacteric fruit which has certain characteristic physical stages while maturing due to the production of ethylene. Date fruit ripening is associated with four maturation stages that are generally recognized using Arabic terms. An immature fruit is having a hard texture and green color and is considered at Kimri stage of it its maturity. A hard, yellow, and edible Barhi date is considered to be in the Khalal stage; a soft, brown, and semi-ripe stage reflects the Rutab stage of dates and a final soft, dark brown, and fully ripe date fruit is considered to reflect the Tamar stage [2]. These different phases of date fruit maturation are also characterized by their specific textural, sensory, and chemical characteristics [3,4]. Date fruits are consumed in different stages of maturity, such as Khalal, Rutab, and Tamer stages, due to certain organoleptic characteristics and consumer preferences [5]. Barhi is a seasonal date and is cherished when at its Khalal stage (sweet taste and yellow color). Barhi dates have a short span at this stage after harvest (August–October in Saudi Arabia) and change to a Rutab stage quickly (approximately 1 week) if not preserved properly. The Khalal Barhi has high importance from a market and consumer perspectives due to its high demand, characteristic taste, and associated health benefits. These quality attributes of Barhi dates at a Khalal stage may be due to bioactive compounds and certain flavor components. Due to these reasons, it is important to study and establish techniques that can increase the shelf-life of Barhi at the Khalal stage and preserve its characteristics for a longer time [6]. Edible film coating is a developing and frequently used technique in which a thin film layer of edible materials is applied on fruit and vegetable surfaces. Coating can aid in the control of gas exchange and transfer of moisture, thereby modifying the internal atmosphere, maintaining quality, and prolonging the postharvest shelf life of fruit and vegetables [7,8]. There are different types of materials for such coatings, including biodegradable polymers such as polysaccharides, lipids, and proteins [9]. Chitosan is a polysaccharide material, derived from nature, that is non-toxic, biodegradable, biocompatible, and antimicrobial, and has good film-forming properties; thus, it is often used as coating material [10]. Generally, natural products extracted from different types of plant by-products contain phenolic substances with beneficial bioactivities, such as antimicrobial and antioxidant activity, and hence, there is ongoing research on the use of these phytochemicals into chitosan films and coating them on fresh agro-produce [11]. The current study was carried out to investigate the effect of edible chitosan coatings with natural functional ingredients obtained from orange peel and olive cake on the quality attributes of fresh Barhi date fruit. Evaluation of different physicochemical attributes of Barhi dates (coated or uncoated) along with characterization of surface structure using electron microscopy were also carried out.

## 2. Materials and Methods

### 2.1. Materials

Fresh Barhi dates were obtained from local dealers in Riyadh and Qassim provinces during August–October 2021, and various trials were immediately carried out to date samples. The fruit samples were identified by experts in the College of Food and Agriculture for the appropriate maturity at Khalal stage and then properly labeled. Fruits were inspected and any damaged fruits were removed from the spikelet and only good quality fruits on the spikelet were selected for further treatment and experiment. Fresh olives were obtained from the local market and the oil was extracted using mechanical oil extractor (SUS 304, SUS Machines, Shanghai, China) using the cold pressing technique. The remaining cake material was dried and ground to powder form (moisture 11.32 ± 1.43%). Orange peels were manually removed from the fresh oranges obtained from the local farm. Orange peels were dried, and then ground to make the powder form (moisture 7.67 ± 1.85%). Both olive cake and orange peel powders were stored at 4 °C before further use. All chemicals used were of reagent grade.

### 2.2. By-Product Extracts’ Preparation

A known weight of olives cake and orange peel powders (50 g) was extracted in distilled water at 70 °C for 30 min followed by cooling at room temperature and filtration to remove impurities. The filtrate was dried under vacuum at 50 °C using a rotary evaporator and the extract was saved in sterilized bags and stored at −20 °C before use for physico-chemical analysis. The extraction process was repeated for the filtered residue and substantial quantities of both olive cakes extract (OCE) and orange peel extract (OPE) were obtained. The extraction process was carried out at a low temperature to preserve the phytochemical compounds in the extract [12,13].

### 2.3. Preparation of Coating Solution and Application

A stock solution of chitosan was prepared by dissolution of chitosan powder (2% *w*/*v*) 1% acetic acid along with 1% glycerol as a plasticizer. The OCE and OPE were added to the chitosan solution at the ratios of 1% and 2.0%, mixed and homogenized in a blender (Acapulco 30564, Palson Co., Kunshan, China) for 5 min until a smooth solution was obtained [14]. Fresh Barhi date fruit was washed and allowed to dry at room temperature. Once completely dried, each spikelet of date fruit was dipped in various coating solutions for 5 min. Spikelets were carefully removed from the coating solution and kept on a sieved stand to allow drying at room temperature for 2–3 h. A small plastic fan (CK2215, Clickon, Liwan, China) was also used to allow quicker drying of the coated dates. Afterward, dates were removed from the spikelet and grouped into six batches:

- Batch A: Uncoated as a control;
- Batch B: Coated with chitosan (2%) solution;
- Batch C: Coated with chitosan (2%) and OCE (1%) solution;
- Batch D: Coated with chitosan (2%) and OCE (2%) solution;
- Batch E: Coated with chitosan (2%) and OPE (1%) solution;
- Batch F: Coated with chitosan (2%) and OPE (2%) solution.

Each batch was then packed in a polyethylene plastic container with 5–6 holes in the lid and stored at 4 ° C for different periods (0, 7, 14, 21, and 28 days). Each batch was prepared in triplicates.

### 2.4. Physicochemical Characterization

The moisture content was estimated using the oven drying method [15]. Water activity was measured using a water activity meter (Aqualab CX3-TE, Labo-Scientifica, Parma, Italy). After equilibration, the water activity value was recorded. The total soluble solids (TSS) contents of the samples, either uncoated or coated (°Brix) were determined a 20 °C using a digital refractometer (DR 6000, A. Kruss Optronic GmbH, Hamburg, Germany), and the °Brix value was calculated using the dilution factor. The pH determination included homogenization of 5 g of sample with 50 mL of deionized distilled water followed by pH measurements using a Corning 240 pH meter (Corning Scientific Products, New York, NY, USA). A potentiometric titration method [15] using NaOH solution (0.1 N) with pH 8.1 was applied to measure the titratable acidity of the slurry made from date samples.

### 2.5. Microstructure Determination Using Scanning Electron Microscopy (SEM)

In order to carry out the SEM study, small cuttings of date fruit surfaces (coated and uncoated) were obtained and freeze dried for 3 days. The surface morphology of coated and uncoated date skin samples was studied and scanned using a field-emission scanning electron microscope (JSM-7600, Jeol Ltd., Tokyo, Japan) at a resolution of 1000 [16]. It was also equipped with energy dispersive spectroscopy or EDS. Previously dried samples were coated with platinum for 35 s and the total layer thickness of the sample was 25 mm to avoid sample charging under the electron beam. The imaging was done using secondary electrons. The working distance was kept 4.5 mm and an accelerating voltage (15 kV) was used during this study. 

### 2.6. Total Phenolics Determination

The total phenolic content of the samples was determined by the method described by Singleton and Rossi [17] using a Folin–Ciocalteu as the main reagent and gallic acid as a standard, and the results were expressed in mg/100 g gallic acid equivalent (GAE).

### 2.7. Antioxidant Activity

The method of Lee et al. [18] was used to evaluate the anti-DPPH radical activities of date fruit samples. One mL of the extract was diluted in methanol, then added mixed with a 2 mL DPPH solution. Methanol was used as a control and the absorbance of the mixtures was read with a spectrophotometer at 517 nm. The DPPH inhibition was estimated using the formula:

$$\mathrm{DPPH}\;\mathrm{inhibition}\;\left(\%\right)=\left(\frac{A_{\mathrm{control}517}-A_{\mathrm{sample}517}}{A_{\mathrm{control}517}}\right)\times 100$$

where A is the absorbance recorded and DPPH inhibition was expressed in percentage.

### 2.8. Color Measurement

The color evaluation of the samples was carried out using instrumental color method before and during storage. A Hunter Lab colorimeter (Model No. Miniscan^®^ XE plus 4500 L, Hunter Associates Laboratory, Inc., Reston, VA, USA) was used for this evaluation as described previously [12].

### 2.9. Texture Measurement

The samples before and after storage were subjected to texture profile analysis using a texture analyzer (Model CT3, Brookfield, Middleboro, MA, USA). The hardness (kg), cohesiveness, and springiness (mm) were measured in triplicate using a two-cycle test. 

### 2.10. Sensory Evaluation

Sensory analysis was carried out on the first day after coating and then after each week of storage. Twenty semi-trained panelists (male, age range 20–35 years old) were recruited for the evaluation of sensory attributes of date fruit samples using a five-point hedonic scale. The serving of the samples was random using coded and panelists evaluated the texture, color, taste, odor, and overall acceptability. The evaluation was based on the scale of 1–5 (1 = extremely dislike, 5 = extremely like). The sensory evaluation studies were carried out in in three sessions for each storage period (0, 7, 14, 21, and 28 days). The scores were for each sample and each session were calculated as means before subjecting to data analysis. A mean score in the range of 3 to 5 was considered acceptable.

### 2.11. Mold Counts

A pour plate method was applied for the total count of molds at 25 °C for 3–5 days using enumeration (Yeast Extract Glucose Chloramphenicol Agar (YGC), Merck, Darmstadt, Germany)**.** Molds were counted aseptically which involved mixing 25 g of the samples with 225 mL sterile Ringer solution in a Stomacher blender for 1 min.

### 2.12. Principal Component Analysis (PCA)

Coating treatment and storage times were evaluated, for their effects and interrelations on the physicochemical properties of Barhi date samples, using principal component analysis techniques in MultiPlot software [19].

### 2.13. Statistical Analysis

The experiments were carried out using a completely randomized block design with six treatments (control, CH, 1% OCE + CH, 2% OCE + CH, 1% OPE + CH, and 2% OPE + CH) in triplicates, and the physicochemical attributes were evaluated on five storage periods (0, 7, 14, 21, and 28 days). The measurements for each quality attribute were carried out in triplicates. The entire blocks were triplicated independently. SAS software (SAS Institute, Inc., Cary, NC, USA) was used to analyze the data obtained using two-way analysis of variance (ANOVA) and Duncan’s Multiple Range Tests. The treatments and storage times were considered as fixed effects and the replications of the experiments as random effects in model studies. The comparison of sensory evaluation scores was carried out between the treatments and storage times. A General Linear Model (GLM) and Duncan’s Multiple Range Test were used for the comparison of means. The data from triplicate measurements were presented as means and standard deviation (SD). The statistical significances were defined at a probability value of ≤0.05.

## 3. Results and Discussion

### 3.1. Changes in Water Activity, Moisture, and Total Soluble Solids of Coated Fresh Barhi Dates

Table 1 shows changes in moisture, water activity, and total soluble solids of fresh Barhi dates coated with chitosan and/or olive cake or orange peel extracts during cold storage (4 °C). The moisture content of all samples remained above 50% irrespective of the storage period and coating treatment, although it decreased significantly (*p* ≤ 0.05) with an increase in storage time. The coating treatments showed variable effects on the prevention of moisture loss from the date surface. According to Iqbal et al. [20], moisture loss was observed in date fruits during storage as they progressed from the Kimri to the Rutab stage. Coatings were more effective in preventing moisture loss from dates at 4 °C, and coatings experienced slightly less moisture loss during storage. Hoa et al. [21] found that hydrophobic coatings were more effective than hydrophilic coatings in slowing the weight loss of mango fruits during storage than hydrophilic coatings, which resulted in weight losses comparable to uncoated controls. The water activity of the samples stored at 4 °C was above 0.900 even after 28 days of storage. There were no insignificant differences among different treatments; however, with time, the water activity of the samples during the first two weeks was slightly high compared to those stored for 3 or 4 weeks. The reduction in water activity of the date samples with time could be due to a reduction in moisture content. Similarly, an increase in TSS was observed with storage time, which can be attributed to the loss in the samples’ moisture content. TSS was varied between coating materials, with 1% OPE + CH coating providing the lowest percentage of TSS and the control sample providing a higher value than the other treatments at week 4. The increase in TSS with storage time implies that the fruits underwent anaerobic respiration, in which simple sugars are broken down into alcohol and acetaldehyde [22], and the value of TSS as a predictor of the transition from Khalal to Rutab is lost. Permeability in some coating materials, which maintain aerobic respiration, was expected. Moreover, the differences between the storage periods studied were large enough to be statistically significant. This increase could be due to the conversion of some insoluble compounds into soluble compounds (for example, protopectin to pectin) or to water loss from fruits. Thompson and Abboodi [23] found that lower moisture content had a positive effect on the TSS percentage. The current findings are consistent with those reported by Abd El-Moneim et al. [24], where it was reported that the soluble solid contents of Zaghloul date palm cv. were the lowest at zero time, and increased consistently with increasing cold storage period up to sixty days.

### 3.2. Changes in pH, Acidity, Total Phenolics, and Antioxidant Activity (DPPH Inhibition) of Coated Fresh Barhi Dates

Table 2 shows changes in pH, acidity, total phenolics, and antioxidant activity of fresh Barhi dates coated with chitosan and/or olive cake or orange peel extracts during cold storage (4 °C). Regardless of coating materials, the results showed that a slight decrease in the pH and increase in acidity was observed. However, when chitosan was mixed with olive cake or orange peel extracts, an increase in pH was observed with a concomitant decrease in acidity regardless of the storage time.

The study’s data revealed that increasing the storage period increased the acidity of Barhi fruits at different rates for all studied treatments, but most of the studied coating treatments had lower decreasing rates compared to the control sample until the fifth week of storage. The findings are consistent with those reported by Abd El-Moneim et al. [24] on Zaghloul date fruits during the orange season. This was most likely since the film formed by materials altered the fruit’s endogenous CO_2_ and O_2_ concentrations, causing ripening to be delayed. The effect of edible coating on acidity loss has been observed in chitosan- and alginate-coated peaches [25], as well as in avocado coated with methylcellulose [26]. Because organic acids are substrates for many reactions during aerobic respiration in plant cells, the effect of coating on acidity retention could be due to the lower respiration rate found in coated fruits.

As shown in Table 2, the phenolic contents of the control dates were lower than that of the coated samples. The rise in phenolic compounds was due to the presence of phenolic compounds in the extracts obtained from olive cake and orange peel, as these by-products are considered to be rich in phenolic compounds. During the first week of storage, the dates coated using OPE had higher total phenolics than other samples. Variations were observed during the storage of the samples; however, the phenolic compounds of Barhi date alone remained lower than in other samples. Some interesting results were obtained for the coated samples. It was observed that the phenolic compounds of date samples coated with OPE decreased slightly with storage although they were initially higher than the samples coated with OCE, which may be attributed to the stability of phenolic compounds in the coating materials, prepared using olive cake phytochemicals. However, as it has been established already that phenolic compounds are important phytochemicals having various health beneficial properties as well as the ability to prevent microbial spoilage, it seems that the use of olive cake and orange peel extracts can significantly increase the contents of these compounds in coated Barhi dates.

Consistent with the results of total phenolic compounds, the radical scavenging activity of uncoated dates was lower than those of chitosan, OCE + chitosan, and OPE + chitosan-coated dates. This is evidence that coating materials used in this study improve the functional properties of Barhi dates. The radical activity of all samples was decreased with the storage time, but coated fruits still exhibited higher radical scavenging activities as compared to control samples. The radical scavenging activity seems to be less affected by the storage temperature although low-temperature storage or refrigeration is recommended for all types of fresh fruits and vegetables. The above reported results were consistent and well correlated in terms of the bioactive compounds of dates and antioxidant activity results similar to other fruits such as grapes [13]. The declining trend in phenols and antioxidant compounds may be attributed to enzymatic oxidation (polyphenol oxidase and peroxidase) during storage [27]. Both OCE and OPE are expected to contribute significantly to the occurrence of various type of antioxidants, phytochemicals and bioactive compounds, which are capable of preserving oxidations reactions, reduce microbial growth, and enhance health benefits, as reported earlier [12,16]

### 3.3. Changes in Color and Texture of Coated Fresh Barhi Dates 

The results of the color are presented in Table 3, where L* indicates whiteness or brightness/darkness, a* redness/greenness, and b* yellowness/blueness. In terms of L* values, it can be observed that the lightness or brightness of the date color decreased with the storage time for both coated and uncoated dates, which indicated that the dates were brighter at the start of the storage time, whereas this decreased with the progression of the storage time. The brightness of the samples on the first day showed insignificant differences; however, a naked eye observation revealed that the dates coated with a mixture of OCE and chitosan were brighter in appearance as compared to the other samples and coated dates. However, this visual difference was not detected during the instrumental measurement of color values.

The a* values for the control sample were decreased with time and fluctuated in treated samples and the differences were varied between treatments. The b* values were decreased with the storage time, particularly on the 28th day of storage, showing a decrease in the yellowness of the dates. The treatment seemed to have invariable effects on the color values; for example, the brightness seemed to be better preserved in coated dates stored at 4 °C. Overall coating with OCE extract has positive effects on the color values of dates. The decrease in b* values indicates that the yellowing of the samples was significantly lower in the coated fruit when compared to the control sample. The lower change in b values was most likely due to the retention of fruit pigments, with Maskan [28] reporting that a lower b* value might be due to the hydrolysis of chlorophyll and carotenoid pigments, non-enzymatic Maillard browning, and formation of brown pigments. Coatings have been found to mitigate the effects of such interactions. It has been reported that changes in the brightness of fruits can be used to predict fruit browning [29]. Surface coating of the fruits with chitosan and/or OCE and OPE reduced the rate of loss in the brightness of the fruits by lowering the rate of loss in L values, and it was discovered that as the concentration of OCE or OPE increased, the rate of loss in L value decreased. The findings revealed that coating preserved the brightness of the fruits by giving them a shiny appearance. According to Eissa [30], chitosan coating reduces oxidative enzyme activity, such as polyphenol-oxidase, peroxidase, catalase, and laccase, which are associated with discoloration. As a result, the changes in color parameters of the control samples in this study were more pronounced than those of the coated samples. In a study by Jiang and Li [31], it was determined that chitosan coating inhibits the growth of some fungi and delays further decay of stored longan fruit. Similarly, chitosan coating appears to reduce the pH of mushrooms during storage, as reported by Eissa [30]. This is an indication that chitosan coating reduces pathogen development and accordingly could be partially useful in delaying discoloration and browning during storage.

The results of textural analyses of the date samples were shown in Table 3. The hardness, cohesiveness, and springiness of date fruits were decreased with storage time with a significant reduction observed in hardness. However, coating of the date samples alleviates the effect of storage on date texture. The loss of firmness is an important criterion that indicates the quality of the date during storage. The chitosan and/or OCE and OPE mixture coating improved the texture of the date significantly. The firmness of all samples decreased with storage, but chitosan and/or OCE and OPE-coated dates retained their firmness longer than the control sample. At the end of the third week, both the control and coated samples experienced rapid firmness loss, with the control samples experiencing the most rapid changes. According to Mannozzi et al. [32], who studied the effect of edible coatings on the quality of blueberry fruits during shelf-life, the higher firmness values of the coated date are likely due to the presence of the coating agent, which provides structural rigidity at the product’s surface. Date softening during storage is determined by cell structure deterioration, cell wall composition, and intracellular materials, as reported for guava by Hong et al. [33]. The firmness of chitosan and/or OCE and OPE-treated dates may be retained due to a reduction in respiration and other maturation processes during storage as a result of covering the date’s cuticle and lentils with the materials’ coating. The observed firmness loss is consistent with the findings of Hong et al. [33], who investigated the effect of chitosan coating on guava.

### 3.4. Sensory Evaluation of Coated Fresh Barhi Dates Samples

Samples were coded anonymously and the fruit was evaluated for texture, color, taste, odor, astringency, and overall acceptability using a five-point hedonic scale, where one denotes ‘‘disliked extremely” and five reflects ‘‘liked extremely”. The results of the sensory evaluation are presented in Table 4. The sensory evaluation data showed non-significant changes in acceptability and sensory properties of the Barhi dates due to different coating materials throughout the storage period. The overall acceptability of control or those coated with 2% OCE or 1% OPE and 2% chitosan were closer to each other. However, the sensory properties of all the samples seemed to get a low score but were not significant with the advent of storage time. Dates stored at 4 °C were fairly acceptable until the end of the storage period and showed fairly high acceptability compared to control samples, revealing a positive effect of coating materials on the color, texture, and sensory quality of Barhi dates. Abu-Shama [34] investigated the effect of edible coatings on the fruit quality of the Barhi date cultivar and concluded that all edible coating treatments studied had little to no effect on the organoleptic characteristics of Barhi date fruits, implying that these treatments could be used to extend the shelf life of Barhi date fruits.

### 3.5. Changes in Molds of Coated Fresh Barhi Dates

**Figure 1:**
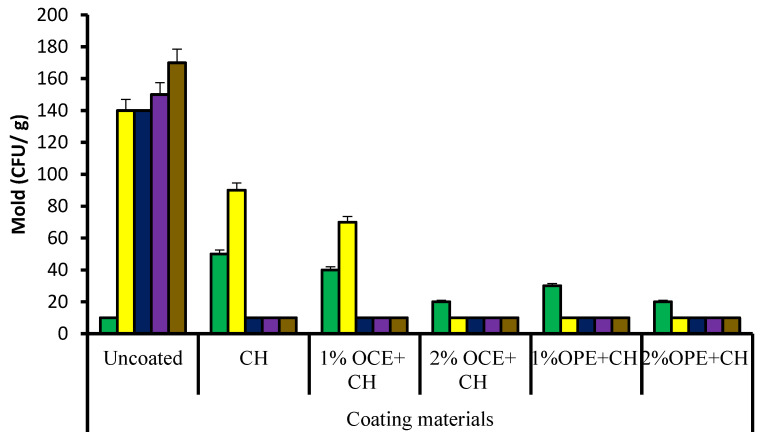
Mold (CFU/g) of fresh Barhi dates coated with chitosan and/or olive cake or orange peel extracts during cold storage (4 °C). CH, Chitosan; OCE, Olive cake extract; OPE, Orange peel extract. Columns from left to right (storage period, days), 0 (green), 7 (yellow), 14 (blue), 21 (purple), and 28 (brown).

Figure 1 shows molds (cfu/g) of fresh Barhi dates coated with chitosan and/or olive cake or orange peel extracts during cold storage (4 °C). There was a significant increase in the fungal content of the control dates during storage. However, in chitosan-coated date molds increased during the first week, and thereafter, dropped significantly, but in other coating materials, they were significantly lower than in the control samples. Molds had a maximum value of 170 cfu/g on day 28 for the control samples, while coated date had a value of 10 cfu/g at the same storage time. The increase in simple sugars and decrease in moisture content of the fruits during date maturation create a better microenvironment for fungal growth [35]. In comparison to the control samples, the results showed that chitosan and/or OCE and OPE-containing coatings performed best at 4 °C in terms of keeping fungal numbers under control. Differences could be attributed to molds’ inability to use sugars as a substrate [35]. Furthermore, it appeared that the preservation of simple sugars in coated samples inhibited fungal growth. According to Lasram et al. [36], all coatings were more effective in retarding fungal growth at 3 °C than at 25 °C because the lower temperature was suboptimal for fungal growth and metabolism.

### 3.6. Surface Characteristics of Coated and Uncoated Date Fruit

**Figure 2:**
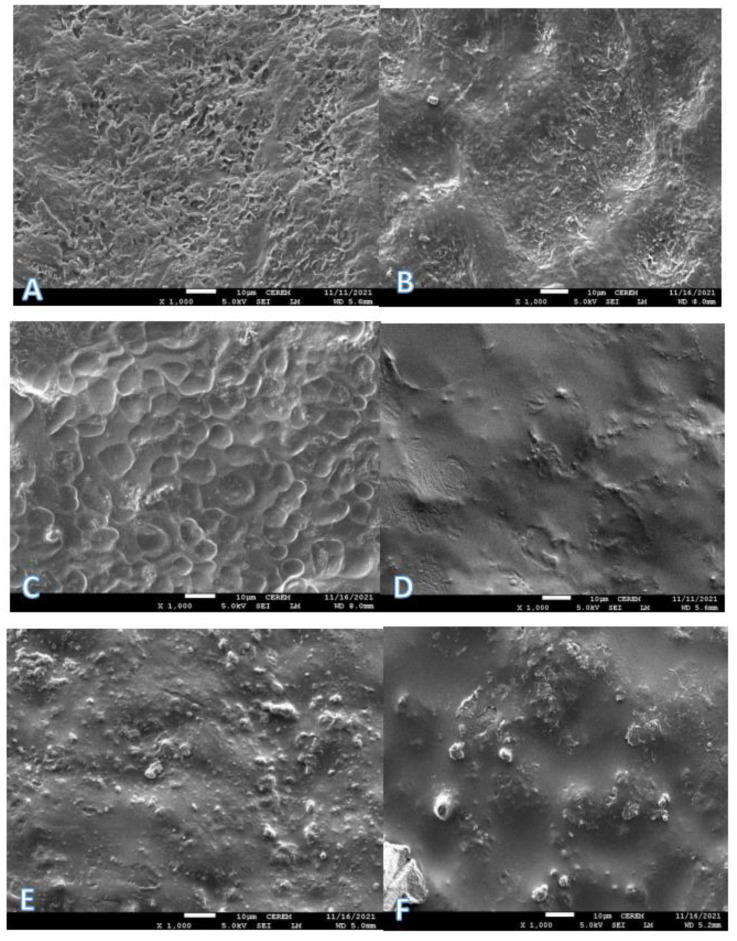
Scanning electron microscopy images (resolution × 1000) of Barhi date surfaces when fresh (**A**) and coated using 2% chitosan (**B**), 1% olive cake extract and 2% chitosan (**C**), 2% olive cake extract and 2% chitosan (**D**), 1% orange peel extract and 2% chitosan (**E**), and 2% orange peel extract and 2% chitosan (**F**).

The surface and cross-section microstructures of coated dates with chitosan (2%) alone, chitosan (2%) and OCE (1–2%), and chitosan (2%) and OPE (1–2%) were examined using a scanning electron microscope and compared with those of uncoated date fruit surfaces. The visualization of the structural characteristics of coated date fruit and that of fresh date fruit surface was carried out for the first time (to the best of our knowledge). Figure 2A shows the scanning electron microscope (SEM) images (resolution ×1000) of fresh Barhi dates (without coating). The fruit surface was rough due to the natural structure of the date fruit surface and can be due to the cellulose and pectin network on the surface of the fruit. These natural openings may be helping in the transfer of gases and may also in some cases aid in the ripening process. Chitosan, which is normally used in the development of edible coatings, was coated alone (2% solution) and the SEM studies of chitosan-coated date fruit surface (Figure 2B) showed that the cracks that appeared naturally on the fruit surfaces were covered and the surface of the fruit became smooth. The coating material (chitosan 2%) was modified with OCE (1–2%) and the SEM studies (Figure 2C,D) showed that the fruit surface became very smooth, particularly when the OCE concentration was 2% in the coating solution. It appears that the OCE 1% chitosan solution might be less viscous than the OCE 2% chitosan solution, which might have completely covered the rough surface structures. SEM studies (Figure 2E,F) of citrus peel extract in edible chitosan coating showed a less smooth surface and some cracks were visible somewhat similar to cracks that appeared when fruits were coated with chitosan alone. Hence, the SEM studies showed that olive cake extract produced the best coating smoothness, and this can be attributed to the presence of certain fatty materials in the extracts that can also help in improving the surface color. Tran et al. [11] also studied the characteristics of the chitosan coating film after the incorporation of plant essential oils and observed that chitosan alone showed a smooth (observed by SEM) and homogeneous surface, whereas essential oils made the membrane less uniform, and the higher the content of essential oil, the less homogeneous the surface was. This might have been due to the addition of essential oils in the membrane matrix, which broke down the continuous structure of the polymer matrix [37]. However, our findings were not in agreement with those of Tran et al. [11], as both the addition of OCE and OPE caused smoothness of the chitosan coating.

### 3.7. Principal Component Analysis (PCA) of Physical Properties of Coated Fresh Barhi Dates

**Figure 3:**
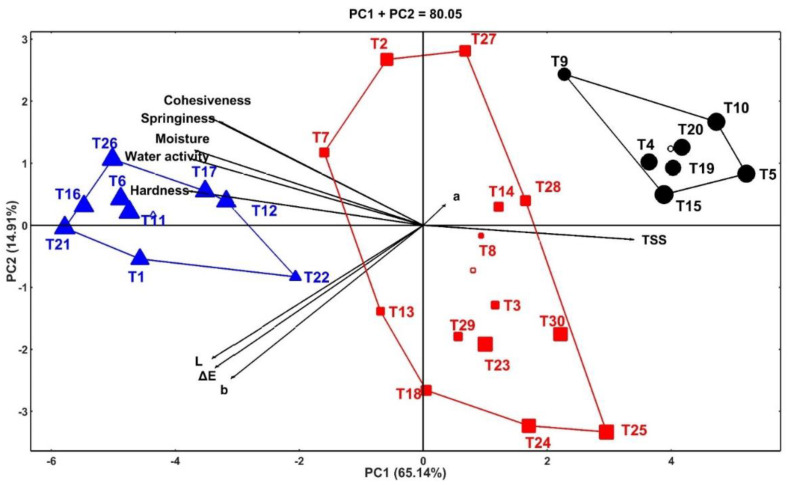
HJ biplot of Barhi dates coated chitosan fortified with 1 and 2 % orange peel extract (OPE) and olive cake extract (OCE). T1, T2, T3, T4, and T5 = negative control (uncoated dates); T6, T7, T8, T9, and T10 = positive control (chitosan-coated dates); T11, T12, T13, T14, and T15 = coated dates fortified with 1% olive cake extract; T16, T17, T18, T19, and T20 = coated dates fortified with 2% olive cake extract; T21, T22, T23, T24, and T25 = coated dates fortified with 1% orange peel extract; T26, T27, T28, T29, and T30 = coated dates fortified with 2% orange peel extract stored for 0, 7, 14, 21, and 28 days, respectively.

To assess the combined effects of treatments on the physical properties of Barhi date, PCA was conducted, and the results are shown in Figure 3. The results indicated a high contribution of the PC1 (65.14%) to the total variability of the plotted components (80.05%) followed by PC2 (14.91%). In the biplot, the cosine of the angle between the vectors of the traits indicated the correlations between them, in which acute, obtuse or straight, or straight angles indicate positive, negative, and no correlations, respectively [38]. Highly positive correlations were seen among color attributes (L, b, and a), moisture and water activity, and texture attributes (hardness, cohesiveness, and springiness), whereas these attributes were negatively correlated with TSS and redness (a). Three clusters of the treatments were seen based on their effect on the physical properties of Barhi dates. The first group (upper right of the graph, black circle symbol) is characterized by a high level of TSS, and this group is composed of the samples untreated (negative control), CH-coated (positive control), and 1 and 2% OCE-coated Barhi dates stored at 4 °C for longer time (21 and 28 days). The high TSS of these samples during prolonged storage indicates rapid decomposition of intact matters of the dates by enzymatic or microbial action and thereby releasing more soluble materials. The second group (right of the graph, red square symbol) is characterized by higher levels of redness (a) than other groups. This group was composed of negative and positive control samples stored at 4 °C for 7 and 14 days, CH-coated with 1% OCE stored at 4 °C for 7 and 14 days, and CH-coated with OPE (1 and 2%) stored at 4 °C for 7, 14, 21, and 28 days. The third group (left of the graph, blue tringle symbol) is characterized by higher levels of color attributes (L, b, and a), moisture, water activity, and texture attributes (hardness, cohesiveness, and springiness) than the other groups. This group was composed of the fresh samples (0 day) of positive and negative controls, and those treated with different concentrations of OCE and OPE indicating that storage at different temperatures and durations adversely affected these attributes. However, the effect of the storage time and temperature were less in the samples treated with OCE and OPE compared to positive and negative controls, suggesting the protective effects of OCE and OPE.

## 4. Conclusions

The current study found that all coating materials increased the shelf life of Barhi date fruits when compared to the control sample. However, when chitosan was combined with OCE and OPE, a pronounced effect was observed. Furthermore, all coating materials increased TSS, were more effective in preventing moisture and firmness loss during storage, did not affect the sensory characteristics of Barhi date fruits, and were extremely effective in preventing fungal growth. SEM image showed that the surface of coated and the uncoated date differed with OCE producing the best smooth coating. Based on these findings, it is possible to conclude that all coating materials tested may be useful in extending the shelf life and maintaining the quality of Barhi date fruits. The PCA analysis showed that the effect of the storage time and temperature were less in the samples treated with OCE and OPE compared to positive and negative controls suggesting the protective effects of OCE and OPE. Further studies demonstrating the effects of storing these fruits at room temperature may also provide valuable information for the preservation of Barhi dates.

## Figures and Tables

**Table 1:** Changes in water activity, moisture, and total soluble solids of fresh Barhi dates coated with chitosan and/or olive cake or orange peel extracts during cold storage (4 °C).

Treatment	Storage Period (Days)				
	0	7	14	21	28
Moisture (%)					
Uncoated	67.72 ± 1.03 aB	67.17 ± 2.01 aA	63.11 ± 1.66 bA	62.36 ± 0.97 cA	61.99 ± 2.88 dB
CH	67.39 ± 2.11 aB	65.56 ± 0.88 bB	63.38 ± 2.03 cA	61.90 ± 1.05 dB	61.67 ± 1.67 dB
1% OCE + CH	66.81 ± 1.55 aB	66.25 ± 0.87 aB	63.17 ± 3.01 bA	62.64 ± 1.44 cA	61.35 ± 1.27 dB
2% OCE + CH	67.89 ± 0.99 aB	66.26 ± 1.01 bB	62.44 ± 1.44 cB	61.63 ± 2.07 dB	62.61 ± 2.04 cA
1% OPE + CH	69.07 ± 1.07 aA	65.26 ± 1.09 bC	62.57 ± 1.62 cB	62.31 ± 2.01 cA	62.01 ± 1.77 cA
2% OPE + CH	67.55 ± 1.23 aB	66.47 ± 2.01 bB	63.03 ± 1.67 cA	62.43 ± 3.07 cA	61.14 ± 2.15 dB
Water activity (aw)					
Uncoated	0.940 ± 0.11	0.928 ± 0.08	0.915 ± 0.06	0.909 ± 0.01	0.905 ± 0.07
CH	0.946 ± 0.12	0.932 ± 0.03	0.911 ± 0.05	0.905 ± 0.04	0.904 ± 0.13
1% OCE + CH	0.934 ± 0.22	0.926 ± 0.04	0.913 ± 0.22	0.916 ± 0.02	0.906 ± 0.23
2% OCE + CH	0.943 ± 0.31	0.938 ± 0.12	0.912 ± 0.34	0.904 ± 0.13	0.901 ± 0.17
1% OPE + CH	0.935 ± 0.07	0.929 ± 0.21	0.911 ± 0.24	0.909 ± 0.16	0.900 ± 0.04
2% OPE + CH	0.940 ± 0.03	0.932 ± 0.09	0.917 ± 0.06	0.909 ± 0.13	0.903 ± 0.07
Total soluble solids (%)					
Uncoated	25.40 ± 1.07 eA	27.20 ± 1.13 dA	29.20 ± 2.02 cA	33.20 ± 2.33 bA	35.80 ± 1.09 aA
CH	25.00 ± 0.98 eA	26.50 ± 1.09 dB	28.34 ± 1.04 cB	29.46 ± 1.23 bB	30.71 ± 2.01 aB
1% OCE + CH	22.20 ± 0.77 dC	22.58 ± 2.01 dE	26.50 ± 1.22 cC	28.45 ± 1.19 bC	29.66 ± 1.67 aC
2% OCE + CH	23.00 ± 0.68 cB	23.64 ± 0.99 cD	25.40 ± 0.88 bD	25.65 ± 2.05 bE	28.90 ± 1.45 aD
1% OPE + CH	21.80 ± 0.69 dC	21.86 ± 0.89 dE	28.76 ± 0.79 cB	29.33 ± 2.07 bB	30.58 ± 1.84 aB
2% OPE + CH	23.40 ± 0.59 eB	24.56 ± 1.05 dC	26.40 ± 0.97 cC	27.66 ± 1.16 bD	28.83 ± 1.58 aD

Values are means of triplicate samples (±SD). Means not sharing common lowercase letters in a row or capital letters in a column are significantly different at *p* ≤ 0.05 as assessed by Duncan’s Multiple Range Test. CH = Chitosan, OCE = Olive cake extract, OPE = Orange peel extract.

**Table 2:** Changes in pH, acidity, total phenolics, and antioxidant activity of fresh Barhi dates coated with chitosan and/or olive cake or orange peel extracts during cold storage (4 °C).

Treatment	Storage Period (Days)				
	0	7	14	21	28
pH					
Uncoated	6.39 ± 0.21 a	6.12 ± 0.82 a	5.89 ± 0.72 ab	5.22 ± 0.41 bBC	4.23 ± 0.63 cB
CH	6.35 ± 0.08 a	6.22 ± 0.67 a	6.13 ± 0.66 a	5.78 ± 0.46 abB	5.12 ± 0.55 bA
1% OCE + CH	6.33 ± 0.11 a	6.39 ± 0.54 a	5.98 ± 0.47 a	5.24 ± 0..91 bAB	5.11 ± 0.71 bA
2% OCE + CH	6.34 ± 0.26 a	6.41 ± 0.44 a	6.12 ± 0.48 a	5.89 ± 0.28 abA	5.69 ± 0.88 bA
1% OPE + CH	6.17 ± 0.56 a	6.52 ± 0.35 a	6.21 ± 0.29 a	6.12 ± 0.51 aA	5.46 ± 0.38 bA
2% OPE + CH	6.34 ± 0.61 a	6.68 ± 0.49 a	6.25 ± 0.34 a	6.12 ± 0.43 aA	5.79 ± 0.39 aA
Titratable acidity (% malic acid)					
Uncoated	0.096 ± 0.002 b	0.172 ± 0.02 abA	0.199 ± 0.012 aA	0.236 ± 0.031 aA	0.257 ± 0.013 aA
CH	0.109 ± 0.01 b	0.131 ± 0.004 bB	0.157 ± 0.013 bB	0.187 ± 0.007 aA	0.203 ± 0.021 aA
1% OCE + CH	0.110 ± 0.02 b	0.129 ± 0.012 bB	0.179 ± 0.006 aA	0.213 ± 0.021 aA	0.232 ± 0.022 aA
2% OCE + CH	0.111 ± 0.003 b	0.125 ± 0.031 bB	0.156 ± 0.003 aB	0.186 ± 0.002 aA	0.202 ± 0.011 aA
1% OPE + CH	0.104 ± 0.001 b	0.130 ± 0.001 aB	0.145 ± 0.005 aB	0.173 ± 0.0024 aA	0.188 ± 0.033 aA
2% OPE + CH	0.104 ± 0.004 b	0.116 ± 0.003 bB	0.114 ± 0.014 aB	0.136 ± 0.0021 aB	0.148 ± 0.002 aB
Total phenolics (mg GAE/g)					
Uncoated	7.18 ± 0.31 aE	5.516 ± 0.12 bF	3.854 ± 0.19 cF	3.458 ± 0.51 cF	2.842 ± 0.11 dF
CH	8.37 ± 0.42 aD	6.705 ± 0.36 bE	5.050 ± 0.21 cE	4.968 ± 0.38 cE	4.125 ± 0.23 dE
1% OCE + CH	9.54 ± 0.45 aC	7.989 ± 0.42 bD	6.442 ± 0.23 cD	5.460 ± 0.46 dD	5.147 ± 0.31 dD
2% OCE + CH	10.19 ± 0.62 aB	8.550 ± 0.45 bC	7.897 ± 0.33 cC	6.987 ± 0.52 dC	6.789 ± 0.41 dC
1% OPE + CH	10.76 ± 0.57 aB	9.408 ± 0.61 bB	8.057 ± 0.37 cB	7.458 ± 0.43 cdB	7.244 ± 0.55 dB
2% OPE + CH	13.10 ± 0.72 aA	11.564 ± 0.39 bA	10.036 ± 0.61 cA	9.546 ± 0.37 cA	9.785 ± 0.67 cA
DPPH inhibition (%)					
Uncoated	43.782 ± 1.22 bD	54.00 ± 0.88 aE	31.69 ± 0.71 cE	15.26 ± 0.95 eF	20.15 ± 0.48 dF
CH	42.760 ± 0.98 bE	54.60 ± 0.78 aE	29.90 ± 0.84 cF	24.25 ± 0.48 dE	22.15 ± 0.39 eE
1% OCE + CH	48.722 ± 2.01 bC	60.65 ± 0.77 aD	38.07 ± 0.87 cD	35.25 ± 0.69 dD	26.25 ± 0.55 eD
2% OCE + CH	78.250 ± 1.34 aA	71.72 ± 0.65 bC	66.25 ± 1.32 cB	55.24 ± 0.75 dC	48.25 ± 0.43 eC
1% OPE + CH	70.102 ± 1.83 bB	82.03 ± 1.03 aB	60.86 ± 1.56 cC	59.58 ± 1.11 dB	58.36 ± 0.57 eB
2% OPE + CH	77.683 ± 0.93 bA	89.61 ± 1.13 aA	65.33 ± 1.72 cA	62.58 ± 2.01 dA	60.45 ± 0.92 eA

Values are means of triplicate samples (±SD). Means not sharing lowercase letters in a row or capital letters in a column are significantly different at *p* ≤ 0.05 as assessed by Duncan’s Multiple Range Test. CH = Chitosan, OCE = Olive cake extract, OPE = Orange peel extract.

**Table 3:** Changes in color and texture of fresh Barhi dates coated with chitosan and/or olive cake or orange peel extracts during cold storage (4 °C).

Treatment	Storage Time														
	0			7			14			21			28		
	L*	a*	b*	L*	a*	b*	L*	a*	b*	L*	a*	b*	L*	a*	b*
Color															
Uncoated	58.76 ± 0.73 aA	1.89 ± 0.04 aA	38.99 ± 1.23 aA	46.01 ± 1.22 cC	1.20 ± 0.03 dA	26.75 ± 1.02 cE	51.14 ± 1.31 bD	0.41 ± 0.01 eB	31.60 ± 1.01 bE	42.44 ± 1.12 dE	1.65 ± 0.07 bA	25.75 ± 1.03 dD	39.19 ± 1.05 eF	1.52 ± 0.03 cA	24.87 ± 1.11 eB
CH	59.27 ± 1.02 aA	1.61 ± 0.12 bA	35.00 ± 1.09 aC	53.05 ± 0.89 bB	0.58 ± 0.05 cB	27.20 ± 1.23 cD	49.66 ± 1.09 cE	0.35 ± 0.04 dB	29.30 ± 0.79 bE	43.87 ± 0.89 dD	2.08 ± 0.14 aA	23.32 ± 0.99 dE	41.25 ± 1.22 eE	1.98 ± 0.12 aA	19.48 ± 0.89 eD
1% OCE + CH	57.54 ± 0.88 aB	0.96 ± 0.34 bB	35.74 ± 2.03 aC	55.88 ± 1.04 bA	1.46 ± 0.06 aA	32.96 ± .96 cC	54.73 ± 0.69 cB	0.53 ± 0.03 cB	34.55 ± 1.03 bB	49.45 ± 0.74 dC	1.47 ± 0.21 aA	28.36 ± 0.79 dC	45.29 ± 1.34 eC	1.65 ± 0.21 aA	22.48 ± 0.78 eC
2% OCE + CH	57.37 ± 0.79 aB	0.62 ± 0.24 cC	37.20 ± 0.89 aB	56.29 ± 0.79 bA	1.22 ± 0.12 aA	33.53 ± 0.55 cB	56.16 ± 067 bA	0.51 ± 0.02 dB	35.05 ± 0.78 bA	42.28 ± 1.08 cE	0.78 ± 0.05 bB	20.67 ± 0.67 dF	42.75 ± 1.52 cD	0.76 ± 0.08 bB	19.56 ± 0.66 eD
1% OPE + CH	59.26 ± 0.67 aA	1.35 ± 0.67 dA	38.56 ± 0.78 aA	55.95 ± 0.81 bA	1.98 ± 0.14 aA	34.37 ± 0.73 bA	53.92 ± 0.55 dC	1.11 ± 0.06 eA	32.77 ± 0.64 cCD	55.53 ± 1.04 bB	1.72 ± 0.08 bB	34.95 ± 0.59 bA	54.26 ± 1.65 cA	1.52 ± 0.05 cA	32.49 ± 1.02 cA
2% OPE + CH	58.05 ± 0.99aA	1.46 ± 0.46 bA	33.23 ± 0.48 aD	43.78 ± 0.69 cD	1.32 ± 0.08 bA	21.51 ± 0.66 dF	47.04 ± 0.63 dF	1.05 ± 0.02 cA	26.63 ± 0.73 cF	56.28 ± 1.07 bA	1.91 ± 0.06 aA	32.64 ± 0.78 bB	50.25 ± 0.98 cB	1.48 ± 0.06 bA	32.85 ± 0.77 bA
Treatment	Texture														
	Ha	Co	Sp.	Ha	Co	Sp.	Ha	Co	Spr	Ha	Co	Sp.	Ha	Co	Sp.
Uncoated	1020.8 ± 5.34 aF	0.83 ± 0.03 a	0.867 ± 0.12 a	262.17 ± 2.11 bF	0.82 ± 0.01 a	0.911 ± 0.07 a	244.33 ± 4.33 cC	0.74 ± 0.02 a	0.80 ± 0.11 b	55.50 ± 1.07 dF	0.77 ± 0.13 a	0.80 ± 0.04 b	39.50 ± 1.02 eE	0.73 ± 0.11 a	0.77 ± 0.13 b
CH	1069.0 ± 3.66 aE	0.85 ± 0.04 a	0.911 ± 0.08 a	649.17 ± 3.14 bB	0.82 ± 0.04 a	0.867 ± 0.05 a	282.33 ± 3.24 cB	0.77 ± 0.04 a	0.83 ± 0.03 a	78.00 ± 0.98 dA	0.82 ± 0.21 a	0.91 ± 0.12 a	47.33 ± 1.23 eC	0.72 ± 0.04 a	0.81 ± 0.09 a
1% OCE + CH	1166.3 ± 7.55 aD	0.84 ± 0.02 a	0.911 ± 0.04 a	662.83 ± 5.34 bA	0.84 ± 0.07 a	0.867 ± 0.03 a	353.17 ± 2.88 cA	0.81 ± 0.07 a	0.83 ± 0.04 a	57.83 ± 0.83 dE	0.79 ± 0.08 a	0.83 ± 0.06 a	46.50 ± 1.63 eD	0.75 ± 0.13 a	0.77 ± 0.12 b
2% OCE + CH	1344.8 ± 8.55 aA	0.84 ± 0.08 a	0.911 ± 0.06 a	540.67 ± 4.38 bC	0.84 ± 0.05 a	0.911 ± 0.05 a	244.83 ± 4.11 cC	0.77 ± 0.05 a	0.77 ± 0.12 b	63.67 ± 0.91 dD	0.72 ± 0.03 a	0.77 ± 0.05 b	51.67 ± 0.89 eA	0.75 ± 0.06 a	0.77 ± 0.21 b
1% OPE +CH	1238.6 ± 8.56 aB	0.85 ± 0.03 a	0.911 ± 0.07 a	450.67 ± 6.12 bD	0.78 ± 0.09 a	0.833 ± 0.06 a	244.17 ± 5.23 cC	0.74 ± 0.11 a	0.81 ± 0.02 a	67.83 ± 0.69 dB	0.69 ± 0.01 b	0.77 ± 0.04 b	47.50 ± 0.79 eC	0.68 ± 0.03 b	0.73 ± 0.05 b
2% OPE + CH	1227.6 ± 7.99 aC	0.87 ± 0.05 a	0.911 ± 0.02 a	425.00 ± 5.26 bE	0.79 ± 0.03 a	0.800 ± 0.09 a	239.33 ± 2.89 cD	0.72 ± 0.08 a	0.83 ± 0.06 a	65.67 ± 0.59 dC	0.76 ± 0.05 a	0.83 ± 0.13 a	48.83 ± 0.46 eB	0.73 ± 0.08 a	0.81 ± 0.16 a

Values are means of triplicate samples (±SD). Means not sharing common lowercase letters in a row or capital letters in a column are significantly different at *p* ≤ 0.05 as assessed by Duncan’s Multiple Range Test. Abbreviations: CH, Chitosan; OCE, Olive cake extract; OPE, Orange peel extract; Ha, hardness; Co, Cohesiveness; Sp., Springiness.

**Table 4:** Sensory evaluation of fresh Barhi dates coated with chitosan and/or olive cake or orange peel extracts during cold storage (4 °C).

Treatment	Storage Period (Days)											
	0						7					
	Texture	Color	Taste	Odor	Astringency	Overall Accept.	Texture	Color	Taste	Odor	Astringency	Overall Accept.
Uncoated	4.17 ± 0.23	4.33 ± 0.32	4.33 ± 0.57	4.25 ± 0.67	4.33 ± 0.28	4.00 ± 0.23	4.25 ± 0.38	3.75 ± 0.22	3.25 ± 0.44	3.50 ± 0.77	3.75 ± 0.38	4.00 ± 0.83
CH	4.50 ± 0.31	4.50 ± 0.35	4.00 ± 0.56	4.50 ± 0.49	4.17 ± 0.47	3.83 ± 0.35	3.75 ± 0.29	3.50 ± 0.76	3.50 ± 0.22	3.75 ± 0.62	3.75 ± 0.42	4.33 ± 0.72
1% OCE + CH	4.17 ± 0.44	4.50 ± 0.62	4.05 ± 0.55	4.58 ± 0.44	4.00 ± 0.51	3.95 ± 0.67	3.75 ± 0.66	3.75 ± 0.61	4.00 ± 0.82	3.50 ± 0.56	3.50 ± 0.83	4.33 ± 0.76
2% OCE + CH	4.00 ± 0.54	4.00 ± 0.47	4.42 ± 0.49	4.50 ± 0.29	3.67 ± 0.47	4.17 ± 0.82	3.75 ± 0.27	4.00 ± 0.53	3.50 ± 0.33	3.25 ± 0.51	4.00 ± 0.91	4.33 ± 0.59
1% OPE + CH	4.00 ± 0.34	3.83 ± 0.56	4.17 ± 0.38	4.05 ± 0.78	3.17 ± 0.66	4.00 ± 0.54	3.50 ± 0.48	3.00 ± 0.19	3.75 ± 0.17	3.50 ± 0.41	3.50 ± 0.36	4.00 ± 0.62
2% OPE + CH	3.83 ± 0.45	3.83 ± 0.33	3.50 ± 0.28	4.00 ± 0.81	3.25 ± 0.43	3.72 ± 0.37	4.00 ± 0.53	3.25 ± 0.26	3.50 ± 0.28	3.75 ± 0.55	3.75 ± 0.47	4.00 ± 0.28
Treatment	14						21					
	Texture	Color	Taste	Odor	Astringency	Overall accept.	Texture	Color	Taste	Odor	Astringency	Overall accept.
Uncoated	3.67 ± 0.24	4.00 ± 0.47	4.00 ± 0.46	4.00 ± 0.83	3.67 ± 0.42	3.63 ± 0.16	3.15 ± 0.41	3.17 ± 0.29	3.14 ± 0.82	3.85 ± 0.22	3.42 ± 0.37	3.16 ± 0.46
CH	3.67 ± 0.66	3.33 ± 0.38	4.00 ± 0.34	3.67 ± 0.46	3.33 ± 0.65	3.63 ± 0.33	3.65 ± 0.72	3.33 ± 0.32	3.48 ± 0.33	3.45 ± 0.53	3.28 ± 0.46	3.48 ± 0.63
1% OCE + CH	3.67 ± 0.37	3.00 ± 0.39	3.67 ± 0.87	3.33 ± 0.36	3.00 ± 0.72	3.25 ± 0.27	3.85 ± 0.82	3.00 ± 0.41	3.58 ± 0.46	3.52 ± 0.61	2.78 ± 0.23	3.75 ± 0.27
2% OCE + CH	3.67 ± 0.57	3.00 ± 0.27	3.00 ± 0.77	3.33 ± 0.33	3.33 ± 0.39	3.13 ± 0.28	3.58 ± 0.88	3.00 ± 0.52	3.24 ± 0.37	3.48 ± 0.72	3.47 ± 0.29	3.65 ± 0.41
1% OPE + CH	4.33 ± 0.88	3.33 ± 0.81	3.33 ± 0.48	3.67 ± 0.87	3.00 ± 0.22	3.63 ± 0.38	4.18 ± 0.92	3.33 ± 0.84	3.45 ± 0.32	3.75 ± 0.54	3.17 ± 0.37	3.88 ± 0.46
2% OPE + CH	3.67 ± 0.89	3.33 ± 0.71	4.00 ± 0.43	3.33 ± 0.67	3.50 ± 0.87	3.63 ± 0.44	3.52 ± 0.26	3.33 ± 0.73	3.78 ± 0.49	3.17 ± 0.29	3.46 ± 0.27	3.63 ± 0.88
Treatment	28											
	Texture	Color	Taste	Odor	Astringency	Overall accept.						
Uncoated	3.25 ± 0.35	3.27 ± 0.43	3.23 ± 0.32	3.75 ± 0.32	3.39 ± 0.32	3.22 ± 0.26						
CH	3.57 ± 0.68	3.29 ± 0.22	3.46 ± 0.23	3.53 ± 0.33	3.32 ± 0.19	3.46 ± 0.37						
1% OCE + CH	3.61 ± 0.48	3.11 ± 0.31	3.61 ± 0.26	3.29 ± 0.53	2.81 ± 0.37	3.47 ± 0.51						
2% OCE + CH	3.57 ± 0.27	3.13 ± 0.32	3.29 ± 0.17	3.51 ± 0.52	3.39 ± 0.41	3.71 ± 0.65						
1% OPE + CH	3.27 ± 0.44	3.39 ± 0.54	3.25 ± 0.52	3.69 ± 0.42	3.27 ± 0.47	3.89 ± 0.33						
2% OPE + CH	3.21 ± 0.63	3.23 ± 0.53	3.68 ± 0.39	3.23 ± 0.43	3.52 ± 0.38	3.74 ± 0.66						

Values are means of triplicate samples (±SD). Means not sharing common lowercase letters in a row or capital letters in a column are significantly different at *p* ≤ 0.05 as assessed by Duncan’s Multiple Range Test. CH, Chitosan; OCE, Olive cake extract; OPE, Orange peel extract.

## Data Availability

The data presented in this study are available in this article.
